# Utilisation of Rosehip Waste Powder as a Functional Ingredient to Enrich Waffle Cones with Fibres, Polyphenols, and Carotenoids

**DOI:** 10.3390/foods14010090

**Published:** 2025-01-01

**Authors:** Alexandra Raluca Borşa (Bogdan), Adriana Păucean, Melinda Fogarasi, Floricuța Ranga, Andrei Borșa, Anda Elena Tanislav, Vlad Mureșan, Cristina Anamaria Semeniuc

**Affiliations:** 1 Department of Food Engineering, University of Agricultural Sciences and Veterinary Medicine of Cluj-Napoca, 3-5 Calea Mănăştur, 400372 Cluj-Napoca, Romania; raluca.borsa@usamvcluj.ro (A.R.B.); adriana.paucean@usamvcluj.ro (A.P.); melinda.fogarasi@usamvcluj.ro (M.F.); andrei.borsa@usamvcluj.ro (A.B.); anda.tanislav@usamvcluj.ro (A.E.T.); vlad.muresan@usamvcluj.ro (V.M.); 2 Department of Food Science, University of Agricultural Sciences and Veterinary Medicine of Cluj-Napoca, 3-5 Calea Mănăştur, 400372 Cluj-Napoca, Romania; floricutza_ro@yahoo.com

**Keywords:** proximate composition, energy value, pH, colour, techno-functional properties, *β*-carotene, lycopene, lutein

## Abstract

The solid waste generated from processing rosehip fruits into jam is valuable due to its rich content in fibres, polyphenols, and carotenoids; it could be valorised as a functional ingredient in a powder form to enrich food products. This study aimed to test its potential as a value-added ingredient, especially to enrich waffle cones with fibres, polyphenols, and carotenoids. In this regard, four formulations of waffle cones were prepared by partially substituting wheat flour with rosehip waste powder at 0%, 10%, 15%, and 20%, reaching concentrations of 0%, 3.7%, 5.7%, and 7.5% of the total batter, respectively. These were assessed for their sensory, textural, and techno-functional properties; proximate composition (including crude fibre); energy value; pH; and colour, as well as the content of carotenoids and polyphenols. The contribution of rosehip powder to the production cost of these waffle cone formulations was also determined. The results showed that using rosehip waste powder as an ingredient reduced the waffle cones powder’s capacity to hold water (from 3.11 g/g to 2.64–3.08 g/g) and to swell (from 4.98 mL/g to 4.23–4.48 mL/g), while it increased their oil-holding capacity (from 0.93 g/g to 0.96–1.19 g/g) and the content in fibre (from 1.58% to 3.41–4.83%), polyphenols (from 400.70 µg/g to 1732.26–2715.69 µg/g), and carotenoids (from n.d. to 6.86–14.28 µg/g); however, the solubility (72.65–75.33%), hardness (2.31–2.83 N), and fracturability (6–8) were not significantly influenced. The sensory acceptability of enriched waffle cones (92–93%) was higher than that of control waffle cones (90%). The production cost of a waffle cone increased by EUR 0.004–0.009 when wheat flour was substituted by rosehip powder in concentrations of 10–20%. In conclusion, to enrich waffle cones with fibres, polyphenols, and carotenoids, at least 10% of wheat flour must be substituted with rosehip waste powder in their manufacturing recipe.

## 1. Introduction

Ice cream cones belong to the rolled sugar waffle category. The batter used for baking them is a fluid mixture made primarily from water, sugar(s), and flour. The sugar content is crucial in achieving the final product’s desired characteristics, with a recommended level between 36% and 48% to roll the waffle discs into cones optimally [1]. During manufacturing, the batter is poured into hinged, two-part baking plates where the flat waffle discs are formed. After baking, these discs are quickly shaped into cones using rolling tools; the sugar recrystallises as the cones cool at room temperature, giving them a hard and glassy texture [2].

Today’s consumers increasingly seek delicious and portable snacks, leading to a remarkable expansion of the global ice cream cones market; valued at USD 2958.15 million in 2023, it is predicted to reach USD 4111.00 million by 2032, achieving a compound annual growth rate of 3.72% from 2024 to 2032. Portability and convenience, innovative flavour combinations, and increasing ice cream consumption are the main elements that drive this market’s growth [3]. Food manufacturers constantly create new waffle cone formulations that improve the overall ice cream experience in response to consumers’ changing preferences and needs. This ongoing innovation in the food industry attracts daring customers and keeps the market dynamic [4]. Increasing awareness regarding health issues and shifting towards healthier eating habits can discourage consumers from purchasing ice cream and, thus, waffle cones [5]. Many brands now provide healthier options, such as gluten-free, low-calorie, or protein-enriched cones, to appeal to health-conscious consumers [3]. Given current consumer preferences for “better-for-me” and “clean-label” foods, manufacturers have discovered that they are a market niche; therefore, using functional ingredients in “less healthy” products can be a healthier option for this category of consumers.

In the food industry, sustainability has become essential, affecting businesses and stakeholders globally [6]. One innovative solution for a more sustainable and circular food value chain is transforming agri-food waste and by-products into value-added ingredients [7]. Numerous ice cream cone producers are already adopting eco-friendly packaging and sourcing ingredients responsibly [3]; this commitment to sustainability resonates with environmentally conscious consumers. One such valuable ingredient could be the solid waste generated while processing rosehip fruits into rosehip purée (a 60% ingredient of the rosehip jam manufacturing recipe). This waste contains nutrients and bioactive compounds such as dietary fibre (79.4%; [8]), unsaturated fatty acids (such as linoleic acid (36–55%; [9]), polyphenols (130.04–207.31 mg/100 g DW; [10]), carotenoids (0.58 mg/100 g; [10]), and minerals (such nitrogen from 19.039 to 28.076 ppm, phosphorous from 553 to 1080 ppm, and potassium from 1142 to 2945 ppm [11]). While it is commonly given to animals as feed, it can also be dried and used as a functional powder to enrich food with these compounds.

In Europe, rosehip fruit processing is mostly centred in Bulgaria, Romania, Sweden, and Hungary, with a capacity of 11,000 tons; 30% of the raw material is wasted during fruit mashing to obtain rosehip purée. Thus, identifying ways to valorise rosehip waste can be an important strategy in a circular economy [12,13]. Evidencing their nutritional compounds and health benefits might encourage researchers and manufacturers to use them in creative products and reduce waste.

Several studies have explored the potential of enriching waffle cones with various ingredients derived from food waste. Austin et al. [14] used Manalagi apple pomace as a source of dietary fibre to improve the nutritional value of waffle cones. Mohd Dom’s study [15] focused on reducing sweet potato peel waste by using its flour as an ingredient in waffle cones. The research conducted by Ashwini et al. [16] tested the suitability of using Musasava banana skin and jackfruit seed flour as substitutes for maida flour in the composition of waffle cones. López-Silva and García-Valle [17] assessed instead the incorporating effect of the spent coffee ground into waffle cones on their quality, sensory properties, and in vitro starch digestibility. Along these lines, fruit and vegetable wastes include a variety of health-promoting compounds and can be used to enhance waffle cones’ functional and nutraceutical qualities.

On the contrary, wheat flour, the main solid ingredient in the waffle cone recipe, is poor in dietary fibre and polyphenols. Waffle cones’ matrix comprises gelatinised starch and denatured proteins, primarily derived from wheat flour. Because of its fibrous and elastic qualities, gluten development affects the handling of batter and disposal of waffle discs from the baking hot plate. That is why adding different ingredients, including those derived from food waste, in the batter of waffle cones reduces the gluten risk in the batter mixture by generating the “dilution” of the gluten. Moreover, protein-rich ingredients detrimentally influence the crispness of waffle cones. Flours with a high water absorption require a lot of water to obtain a smooth batter. As a result, the waffle cones’ textural strength is poor, leading to easy breaking. Starch and gluten mainly contribute to the flour’s water absorption capacity. However, different ingredients rich in proteins and/or fibres could also negatively impact the waffle cones’ quality [1]. Ingredients derived from fruit and vegetable processing waste (pomace, peels, skins, seeds) generally have low protein but high fibre content. However, as studies show, amounts between 5% and 15% of waste ingredients led to waffle cones with good-quality characteristics and improved nutritional properties [14,15,16,17]. Thus, this study aimed to explore the partial substitution of wheat flour with rosehip waste powder to enhance the nutritional profile of waffle cones. Specifically, it investigated the effects of different substitution levels (0%, 10%, 15%, and 20%) on the proximate composition (including crude fibre), energy value, pH, colour, and content in polyphenols and carotenoids of waffle cones, as well as on their sensory, textural, and techno-functional properties; the contribution of rosehip powder to the production cost of these waffle cone formulations was also calculated. This research is the first to use rosehip waste powder to enrich waffle cones with these beneficial compounds.

## 2. Materials and Methods

### 2.1. Preparation of Rosehip Powder

Twelve kilograms of rosehips belonging to the *Rosa canina* L. species were purchased in October 2023 from the Alba Forestry Department of the National Forest Administration-Romsilva (Alba-Iulia, Romania); the fruits were harvested from Romanian spontaneous flora (Aiud and Blaj). Alba County, located in central Romania, spans approximately between latitudes 45°45′ N and 46°30′ N and longitudes 23°15′ E and 24°15′ E. The region features diverse topography, with mountains comprising 61% of the area and hills accounting for 39% [18,19]. The county experiences a temperate continental climate characterised by moderate seasonal variations. The average annual temperature is near 10 °C, and there has been a noticeable upward trend over the past several years due to climate change. With yearly precipitation averaging about 650 mm, the region enjoys warm summers, extended autumns, and mild winters [19,20]. Alba County has a diverse soil composition that includes regosols, aluviosols, luvosols, eroded soils, chernozems, eutric cambisols, and dystric cambisols [21]. The combination of moderate temperatures, adequate rainfall, and fertile soils provides a conducive environment for the growth of wild rosehip (*Rosa canina* L.) [22].

After sorting and cleaning to eliminate spoiled/rotting fruits and physical impurities, they were washed using tap water and strained, allowed to air dry, and weighed. Further, the fruits were cold pressed with an electric oil press machine (PU05; S.C. Jobs Ahead Group, S.R.L., Bucharest, Romania); thus, a rosehip purée (final product) and a solid waste (by-product) were obtained. The solid waste was dried at 40 °C for 24 h in a dehydrator with six trays (DEH-450; Biovita S.R.L., Cluj-Napoca, Romania). Each tray from those six was loaded with 100 g of rosehip waste, resulting in 348 g of dry waste per batch. It was sieved to eliminate allergenic debris, ground with a mechanical mortar (Mortar Grinder RM 200; Retsch GmbH, Haan, Germany) to a fine powder (<10 μm), hermetically sealed in glass jars, and kept in the dark at 4 °C until use. Two batches of rosehip powder were prepared in this manner.

### 2.2. Preparation of Waffle Cones and the Experimental Design

The superior white wheat flour type 000 according to the ash content by Romanian classification (Dobrogea Morărit S.R.L., Constanţa, Romania; moisture 12.4 g/100 g; protein 11.0 g/100 g; carbohydrate 80.0 g/100 g; dietary fibre 1.2 g/100 g; fat 1.0 g/100 g, ash 0.48 g/100 g, and 375.2 kcal/100 g), powdered sugar (Pfeifer & Langen S.R.L., Oradea, Romania), extra-fine iodised sea salt (Carrefour Romania S.A., Bucharest, Romania), refined sunflower oil (Expur S.A., Slobozia, Romania), and sunflower liquid lecithin (Now Health Group, Bloomingdale, IL, USA) were purchased from a local supermarket (Kaufland, Cluj-Napoca, Romania). In addition to these ingredients, tap water and rosehip powder (obtained as described above) were used to prepare four batters according to the recipes in Table 1 (Bc, batter for control waffle cones; B3.7%rp, batter for waffle cones formulated with 3.7% rosehip powder; B5.6%rp, batter for waffle cones formulated with 5.6% rosehip powder; B7.5%rp, batter for waffle cones formulated with 7.5% rosehip powder).

After weighing, the dry ingredients (wheat flour, sugar, salt, and rosehip powder) were mixed with a wire whisk in a stainless-steel bowl, followed by the liquid ingredients (water, oil, and lecithin) until homogenisation. Next, 40 mL of batter was poured onto the hot plate of a waffle cone maker (WAEEHJ1; GGM Gastro International GmbH, Ochtrup, Germany) and baked at 160 °C for 3 min. Each recipe yielded four waffle sheets 14 cm in diameter and 22–23 g, shaped into cones while still hot.

After cooling to room temperature, they were sealed in polyethylene zipper bags (to avoid moisture absorption from the air) and stored in a cool and dry place until testing. Rosehip powder and the four waffle cone formulations were subjected to proximate analysis (content of moisture, protein, fat, ash, total carbohydrate, and crude fibre), calculation of energy value, determination of pH and colour, sensory evaluation (consumer acceptance and quantitative descriptive analysis), instrumental texture analysis, and determination of techno-functional properties as well as polyphenolic and carotenoid compound content.

### 2.3. Analysis Methods

Proximate analysis of rosehip powder and the waffle cone formulations was performed using standardised methods for moisture, protein, fat, and ash [23]; crude fibre was quantified using the Wendee method [24]. The total carbohydrate content (%) was computed as 100-(% moisture+% protein+% fat+% ash) like in Fogarasi et al. [25], and the energy value (kcal/100 g) was calculated as 4 × (g protein + g carbohydrate) + 9 × g fat. The pH of the samples’ aqueous extracts (1 g sample mixed 10 mL distilled water and kept at room temperature for 30 min) was determined using a digital multi-parameter meter (InoLab Multi 9310 IDS; WTW, Weilheim, Germany). The colour attributes of rosehip powder, batters, and waffle cones were measured with a portable colourimeter (NH300; 3NH, Shenzhen, China; D65 illuminant; Φ 8 mm measuring aperture; 10° standard observer) using the CIE L*a*b* colour scheme, as detailed by Socaciu et al. [26].

Consumer acceptance analysis involved the 9-point Hedonic test use (1, dislike extremely; 5, neither like nor dislike; 9, like extremely) to assess the appearance, colour, odour, taste, texture, and overall acceptability of waffle cone formulations; the overall score (scores’ mean of a formulation) and acceptance rate (the mean score of a formulation multiplied by 100 then divided to the maximum score received by it) were calculated for each formulation to determine the preferred one. For the quantitative descriptive analysis of waffle cone formulations, the intensity rating method was applied using a 5-point scale; attributes like fracturability (1, non-friable; 2, slightly friable; 3, friable; 4, very friable; 5, extremely friable), fruity aroma (1, non-fruity; 2, slightly fruity; 3, fruity; 4, very fruity; 5, extremely fruity), hardness (1, soft; 2, slightly soft; 3, hard; 4, very hard; 5, extremely hard), and crispness (1, non-crispy; 2, slightly crispy; 3, crispy; 4, very crispy; 5, extremely crispy) were evaluated for each formulation [14].

The sensory evaluation was carried out in the Laboratory of Sensory Analysis within the Faculty of Food Science and Technology of the University of Agricultural Sciences and Veterinary Medicine of Cluj-Napoca by 25 semi-trained panellists (15 women and 10 men, with an average age of 42 years), teaching staff, and researchers of this faculty. The four waffle cones were randomly placed on white porcelain plates, marked with 3-digit numbers and given to the panellists for assessment in individual cabins. The sensory session was conducted under natural light at a controlled temperature (20 °C). Panellists were instructed to abstain from drinking, eating, and smoking for at least one hour before sensory testing the waffle cone formulations and to drink water between samples to clear their palates.

A CT3 texture analyser (Brookfield Engineering Laboratories, Middleboro, MA, USA) was used for the texture analysis of waffle cone samples, according to the method published by Antonic et al. [27]. The test was performed on 65 mm diameter unrolled waffle cone discs (prepared from 3 mL batter (1.73 g) baked for 1 min at 160 °C); their hardness (N) and fracturability (the number of fractures) were determined by compression using a TA18 sphere probe (12.7 mm diameter) with a speed of 0.5 mm/s and a target of 5 mm. The analysis was performed at room temperature in triplicate.

The techno-functional properties of rosehip powder and the waffle cone formulations were determined using the methods of Jurevičiūtė et al. [28] for water-holding capacity (WCH), swelling capacity (SC), and oil-holding capacity (OHC) or Jeong et al. [29] for solubility.

For the quantification of polyphenolic compounds in rosehip powder and waffle cones, they were extracted from a sample (0.5 g) with a mixture of methanol and water [70% (*v*/*v*); 5 mL], as described by Fogarasi et al. [25]. The extracts thus obtained were chromatographically analysed using the HPLC-DAD-ESI/MS method of Fogarasi et al. [30] on an Agilent 1200 HPLC system (Palo Alto, CA, USA). In this regard, calibration curves were made by injecting five different concentrations of standard substances dissolved in methanol: gallic acid (5–100 µg/mL; R^2^ = 0.9978; LOD = 0.35 μg/mL; LOQ = 1.05 μg/mL), catechin (10–200 µg/mL; R^2^ = 0.9985; LOD = 0.18 μg/mL, LOQ = 0.72 μg/mL), chlorogenic acid (10–50 µg/mL; R^2^ = 0.9937; LOD = 0.41 μg/mL; LOQ = 1.64 μg/mL), cyanidin (10–100 µg/mL; R^2^ = 0.9951; LOD = 0.36 μg/mL, LOQ = 1.44 μg/mL), and rutin (10–100 µg/mL; R^2^ = 0.9981; LOD = 0.21 μg/mL; LOQ = 0.84 μg/mL).

To estimate the content of carotenoid compounds in rosehip powder and waffle cones, they were extracted from a sample (1.0 g) with a mixture of methanol, ethyl acetate, and petroleum ether [1:1:1 (*v*/*v*/*v*); 10 mL], as described by Szabo et al. [31]; the extracts thus obtained were chromatographically analysed using their HPLC-DAD method on the same HPLC equipment. In this regard, calibration curves were made by injecting five different concentrations of standard substances dissolved in ethyl acetate: lutein (1–10 µg/mL; R^2^ = 0.9450; LOD = 0.12 μg/mL; LOQ = 0.36 μg/mL), lycopene (10–50 µg/mL; R^2^ = 0.9976; LOD = 0.22 μg/mL; LOQ = 0.68 μg/mL), and *β*-carotene (1–25 µg/mL; R^2^ = 0.9931; LOD = 0.19 μg/mL; LOQ = 0.58 μg/mL).

### 2.4. Statistical Analysis

The effect of wheat flour partial replacement with powder from rosehip waste in the recipe of waffle cones was determined using one-way ANOVA with Tukey’s post hoc test at a confidence level (*p* < 0.05) of 95%; the Minitab statistical software (version 19.1.1; LEAD Technologies, Inc., Charlotte, NC, USA) was employed in this case.

## 3. Results and Discussion

### 3.1. Physicochemical Properties of Rosehip Powder, Batters, and Waffle Cones

Table 2 shows the chemical composition of rosehip powder used as an ingredient in waffle cone batter. It was characterised by a high carbohydrate content, 79.27%, with 37.70% of this total being crude fibre; the powder had a moisture content of 7.13%, protein of 6.98%, fat and ash of 3.31%, and an energy value of 375 kcal/100 g. Due to its acidic pH (3.73), the rosehip powder caused a significant decrease in the batter’s pH (from 5.70% in Bc to 4.56% in B3.7%rp) and waffle cones’ pH (from 5.57% in WCc to 4.90% in WC3.7%rp). Significant pH differences were also found between the enriched waffle cones, greater with the higher rosehip powder concentration.

Waffle cones prepared by partially substituting wheat flour with rosehip powder had a higher moisture content (2.06–2.43%) than the control ones (1.94%), with significant differences starting from the concentration of 5.6%. Given that the rosehip powder was more concentrated (92.9% dry matter) than the wheat flour (87.6%), the lower dry matter content in enriched waffle cones was due to the pectic substances in rosehips; they bound the water in the batter, preventing its evaporation from the batter during the baking process [32]. This phenomenon also explains the significantly higher total carbohydrate content in waffle cones with rosehip powder (81.05–81.64%) compared to the control ones (78.46%), considering that their moisture contents were comparable (80.0% in wheat flour and 79.3% in rosehip powder). The wheat flour used to prepare waffle cones was rich in carbohydrates but low in dietary fibre. Substituting it partially with rosehip powder resulted in a fibre enrichment effect on waffle cones; the crude fibre content was 2.1–3.0 times higher in waffle cones with rosehip powder (3.41–4.83%), significantly higher than in control ones (1.58%). According to European rules, food may only be claimed to be a source of fibre if it contains at least 3 grammes per 100 grammes [33]. Therefore, the waffle cones prepared with rosehip powder meet this nutritional requirement.

Using rosehip powder as an ingredient in the manufacturing recipe of waffle cones determined a decrease in their protein content (from 14.34% in WCc to 10.83–9.76% in waffle cones with powder) but an increase in fat and ash content (from 4.64% fat in WCc to 5.22–5.79% and from 0.62% ash in WCc to 0.81–0.97%). These changes were due to the lower protein (7.0%) and higher fat (3.3%) and ash (3.3%) content in the rosehip powder than in the substituting flour (11.0% protein, 1.0 fat, and 0.48% ash). It is worth mentioning that the higher fat content comes from the rosehip seeds that contain high amounts of polyunsaturated fatty acids, especially α-linolenic acid and linoleic acid [34]; however, the energy value of waffle cones was not significantly affected. According to the Food Standard Agency, solid food with a fat content of less than or equal to 3 grammes per 100 grammes is considered low fat, while those with more or equal to 20 grammes per 100 grammes is considerede high-fat food [35]. Since the fat content of waffle cones prepared with rosehip powder falls between these limits, they are categorised as medium-fat food.

Other researchers have investigated the effect of rosehip powder on bread quality. For instance, Bryksina et al. [36] noticed increases in bread’s moisture content and acidity with the amount of rosehip powder added (1, 3, 5, and 7% in the final product); their findings align with ours. In a different study, Vartolomei and Turtoi [37] substituted wheat flour with rosehip powder at up to 2.5% concentrations and found no significant effect on the bread’s physicochemical properties. However, Gül and Şen [8] reported that enriching bread with dietary fibre became notable when rosehip powder was used in concentrations greater than 5%.

### 3.2. Colour Properties of Rosehip Powder, Batters, and Waffle Cones

Table 3 centralises the recorded values for the colour attributes of rosehip powder, batters, and waffle cones. The results showed a red-orange colour (h* = 1.05) of moderate intensity (C* = 35.77) for rosehip powder, determined by a specific combination of lightness (L* = 62.02), red hue (a* = 17.75), and yellow hue (b* = 31.06). Its use in the waffle cones manufacturing recipe at concentrations of 3.7–7.5% resulted in an obvious total colour difference between these batters (ΔE* = 16.75–23.05) and the control batter. The red and yellow hues intensified, and the lightness of the batter faded with the amount of wheat flour substituted by rosehip powder; all these changes led to a significant increase in the batter’s colour intensity.

Regarding the waffle cones, the total colour difference between those with rosehip powder and those without (WCc) was large for WC3.7%rp, while it was obvious for WC5.6%rp and WC7.5%rp. Increasing the concentration of rosehip powder in waffle cone formulation significantly reduced their lightness and yellow hue while having a non-significant effect on their red hue; consequently, the colour intensity of the waffle cones was affected considerably. During the baking process of batter, Maillard and caramelisation reactions occurred [8], which determined the formation of a yellow-brown colour in waffle cones. As the amount of rosehip powder used in waffle cones increased, their colour intensified due to the carotenoid pigments in the powder [38]. In contrast to our findings, Gül and Şen [8] noticed an intensification of bread’s red and yellow hues with increasing the concentration of rosehip powder in it; these differences derive from the fact that their bread was baked in hot air, while our waffle cones were baked on a hot plate.

### 3.3. Sensory and Texture Properties of Waffle Cones

The sensory analysis of waffle cones included the Hedonic test (using a nine-point scale) to assess consumer acceptability and the descriptive rating test (using a five-point scale) to quantify the intensity of various sensory attributes. The scores received by the four waffle cone formulations are shown in Table 4 and Table 5; they were between 8.2 and 8.5 for appearance, 8.1 and 8.4 for colour, 7.8 and 8.4 for odour, 8.1 and 8.3 for taste, 8.3 and 8.4 for texture, and 8.1 and 8.5 for overall acceptability, with non-significant differences between them. WC3.7%rp and WC5.6%rp recorded the highest overall scores (8.4 points), followed by WC7.5%rp (8.3 points) and WCc (8.1 points). As a result of these findings, the acceptance rates of waffle cones with rosehip powder (93% for WC3.7%rp and WC5.6%rp; 92% for WC7.5%rp) were higher than that of control waffle cones (90% for WCc). Unlike our results, Chochkov et al. [39] reported a decline in bread’s appearance as the amount of rosehip powder in its formulation increased, while scores for taste improved. In the study of Gül and Şen [8], the overall acceptability of bread formulated with rosehip powder decreased with the substitution level of wheat flour.

The sensory evaluation results of the waffle cones’ texture and aroma attributes revealed non-significant differences among the four formulations in fracturability (3.8–4.0 points), hardness (2.8–3.2 points), and crispness (3.9–4.0 points). Interestingly, the panellists framed the control sample between non-fruity and slightly fruity (1.6 points) and the samples with rosehip powder between fruity and very fruity (3.1–3.6 points). The differences in scores were significant between the samples with rosehip powder and the control one, but not between them. Our findings are in agreement with those of Austin et al. [14], who reported that partially substituting wheat flour with apple pomace powder—up to 70%—had a non-significant impact on the sensory scores of waffle cones in terms of fruity aroma, hardness, and crispness.

The instrumental analysis results of the waffle cones’ textural attributes (Table 6) correlate with those of sensory evaluation, as no significant differences were found among the four waffle cone formulations in terms of hardness (2.31–2.83 N) and fracturability (6–8 fractures). Austin et al. [14] found that the textural properties of control waffle cones can be preserved by substituting up to 50% wheat flour with apple pomace powder.

### 3.4. Techno-Functional Properties of Rosehip and Waffle Cone Powders

Table 7 shows the techno-functional properties of rosehip powder and the powders obtained by grinding waffle cones. Since ice cream contains both water and cream, information such as the water-holding capacity, swelling capacity, oil-holding capacity, and solubility of waffle cones provides evidence of their resistance to end-use. The waffle cone’s WHC decreased with the amount of Rp used at its formulation, from a level of 3.08 g/g in WC3.7%rp to 2.64 g/g in WC7.5%rp, because the WHC of Rp was much lower (1.84 g/g) than that of WCc (3.11 g/g); the difference was significant only at the maximum concentration used. WHC refers to the water amount a sample retains due to chemical or electrostatic interactions within it, being closely linked to the hydration degree of proteins and other polar compounds as well as their hydrogen-bond-mediated hydrophilic interactions [40]; it is positively correlated with the protein and fibre content of a sample [37,40]. Since substituting wheat flour with Rp determined a decrease in the waffle cone’s protein content and increased that of crude fibre, the improvement of its WHC is attributed to the latter; its rise with the increase in fibre content was probably caused by the hydroxyl groups in the chemical structure of fibres, which facilitate water to bind through hydrogen bonds [37]. Vartolomei and Turtoi [37] studied some mixtures of wheat flour and Rp in different ratios, and they also noticed a decline in protein content with increasing Rp addition.

As the swelling capacity of a product is influenced by its water-holding capacity, a downward trend we also noticed was for the waffle cone’s SC with the amount of substituted wheat flour (from 4.48 mL/g in WC3.7%rp to 4.23 mL/g in WC7.5%rp), the SC of Rp (3.74 mL/g) also being lower than that of WCc (4.23 mL/g). In contrast, the waffle cone’s solubility was not significantly influenced by using Rp at its formulation, in a concentration of up to 7.5%, as the Rp’s solubility (73.61%) was close to that of WCc (75.33%); it ranged between 74.15% in WC3.7%rp and 72.65% in WC7.5%rp.

The OHC of flour used to prepare a batter influences its rheology and, therefore, the quality of the finished product [40]. This techno-functional parameter of Rp (1.07 g/g) was slightly greater than that of WCc (0.93 g/g); hence, the waffle cone’s OHC has been trending upward with the amount of Rp used in its formulating (from 0.96 g/g in WC3.7%rp to 1.19 g/g in WC7.5%rp), attributed to the increasing content of fibres in the sample.

### 3.5. Content of Polyphenolic and Carotenoid Compounds in Rosehip Powder and Waffle Cones

Table 8 and Table 9 display the chromatographic analysis results for waffle formulations’ polyphenol and carotenoid content. The total content of polyphenolic compounds in rosehip powder was 7585.05 µg/g, of which 4919.67 µg/g was flavonoids (4228.24 µg/g flavanols, 659.34 µg/g flavonols, and 32.10 µg/g anthocyanins) and 2665.38 µg/g was phenolic acids (2581.46 µg/g hydroxybenzoic acids and 83.92 µg/g hydroxycinnamic acids). Nineteen polyphenolic compounds were identified in rosehip powder, the majority being 2-hydroxybenzoic acid (1245.87 µg/g), catechin (1031.03 µg/g), procyanidin dimer B3 (925.70 µg/g), procyanidin trimer C2 (745.02 µg/g), protocatechuic acid (581.09 µg/g), procyanidin dimer B1 (548.05 µg/g), and procyanidin trimer C1 (459.83 µg/g). Goztepe et al. [41] found catechin (1050 µg/g) as the main compound in their rosehip powder; the other five polyphenolic compounds they identified ranged between 145 µg/g and 279 µg/g. Ghendov-Mosanu et al. [32] instead reported a profile of polyphenolic compounds dominated by procyanidin dimer B1 (2910 µg/g) out of the 15 they found (0.1–105 µg/g) in rosehip powder.

Substituting wheat flour with rosehip powder at 10% and 20% increased the total content of polyphenols by 4.3 to 6.8 times. Three polyphenolic compounds (2-hydroxybenzoic acid, protocatechuic acid, and chlorogenic acid) were found in WCc, while fourteen (in WC3.7%rp) and fifteen (in WC5.6%rp and WC7.5%rp), respectively, were found in the enriched formulations, even though nineteen were present in the rosehip powder. The four polyphenolic compounds present in rosehip powder but absent in waffle cones (procyanidin dimer B3, cyanidin 3-*O*-glucoside, procyanidin dimer B2, and ellagic acid) were likely degraded during the batter baking. This could have been due to the direct interaction of polyphenols with intermediates (such as carbonyl-containing compounds) during thermal treatment, which decreases the total phenolic content via pathways of Maillard reactions, lipid oxidation, and sugar condensation [42].

The chlorogenic acid content in waffle cones significantly decreased with the substituted wheat flour amount, as this constituent was not present in rosehip powder. The polyphenol-enrichment effect of waffle cones resulted from the significant increase in the content of fourteen other polyphenolic compounds, primarily including the 2-hydroxybenzoic acid, catechin, procyanidin trimer C2, protocatechuic acid, procyanidin dimer B1, and procyanidin trimer C1. Only phenolic acids (400.70 µg/g) were identified in WCc, including 312.66 µg/g hydroxybenzoic acids and 88.04 µg/g hydroxycinnamic acids. The flavonoids in the waffle cones with rosehip powder ranged between 1042.99 µg/g and 1788.46 µg/g, while the phenolic acids, between 689.28 µg/g and 927.24 µg/g.

The total content of carotenoid compounds in rosehip powder was 109.04 µg/g, of which 1.50 µg/g was lutein, 31.95 µg/g lycopene, and 75.59 µg/g *β*-carotene. Unlike in our findings, Ghendov-Mosanu et al. [32] did not detect lutein in their rosehip powder; they reported a content of 3.80 µg/g cis-lycopene, 27.20 µg/g all-trans-lycopene, 4.50 µg/g *cis*-*β*-carotene, and 18.40 µg/g all-*trans*-*β*-carotene. Partially substituting wheat flour with rosehip powder in waffle cones had an enrichment effect with carotenoid compounds, as none of the three investigated carotenoids were identified in the control waffle cones. The lycopene content in the waffle cones with rosehip powder varied from 4.17 µg/g to 7.61 µg/g, and that of *β*-carotene from 2.69 µg/g to 6.53 µg/g, determining a total content of these compounds between 6.86 µg/g and 14.28 µg/g.

### 3.6. Cost Analysis of Waffle Cones

To determine to what extent partially substituting wheat flour with rosehip powder influences the price of a waffle cone, we performed a cost analysis considering only raw materials as inputs (see Table 10). The production cost of rosehip waste powder obtained through dehydration was calculated using specific technical and economic parameters, considering a comprehensive material and energy balance analysis. The experimentally determined yield indicates that 348 g of dried waste powder can be produced from 600 g of rosehip waste, corresponding to an approximate yield of 58.1%. Producing one kilogram of powder through dehydration requires 1.72 kg of rosehip waste, with an estimated acquisition cost of EUR 0.2 per kilogram, including gas and labour expenses. The breakdown of operational cost for Rp production presented in Table S1 encompasses equipment depreciation, energy consumption based on the dryer’s power and processing duration, and the energy usage of machines involved in sieving and grinding. Expenses with additional components include labour and maintenance costs related to equipment servicing and auxiliary materials.

Table 10 details the costs of raw materials (EUR) per batch and waffle cone. For the rosehip powder, the calculated cost was EUR 3.01 per kilogram. At substitution levels between 10% and 20%, the raw material costs for the waffle cones with rosehip powder ranged from EUR 0.057 to EUR 0.062, while the raw material cost for the control waffle cone was EUR 0.053. The increase in production costs ranged from 7.55% to 16.98%, depending on the amount of rosehip waste powder used. While moderate, this cost increase is meaningful and should be assessed alongside potential product differentiation, market positioning, and profit margin strategies to determine its overall business impact. It is important to note that the study was conducted under lab-scale production conditions, where costs are typically higher due to limited processing capacity, reliance on manual labour, and the lack of bulk purchasing advantages. Transitioning to industrial-scale production could reduce these costs significantly, potentially halving the current production costs through economies of scale, process automation, and optimised resource utilisation.

## 4. Conclusions

Substituting 10% to 20% of wheat flour with rosehip powder (with final concentrations of 3.7–7.5% of the total batter) increased waffle cones’ moisture, fat, ash, and carbohydrate content while decreasing their protein and pH without significantly affecting their energy value. The higher the percentage of rosehip powder in the waffle cone formulation, the darker their yellow-brown colour became. Due to the content of fibres, polyphenols, and carotenoids in rosehip powder, its use in concentrations between 3.7% and 7.5% in the batter enriched the waffle cones with these constituents. Using rosehip powder to formulate waffle cones diminished their water-holding and swelling capacity while enhancing their oil-holding capacity. However, it did not significantly change their solubility, hardness, and fracturability while increasing their sensory acceptability. As expected, the raw material cost of waffle cones was higher for the enriched formulations but moderate.

Given the anticipated growth of the waffle cone market and producers’ and consumers’ concerns regarding food waste and sustainability, this study’s findings could benefit all stakeholders. Additionally, a marketing study focused on consumers’ perception of this product could be of interest to future research.

## Figures and Tables

**Table 1:** Ingredients used to prepare the waffle cone formulations.

Ingredient	WCc		WC3.7%rp		WC5.6%rp		WC7.5%rp	
	g	%	g	%	g	%	g	%
Water	120	44.8	120	44.8	120	44.8	120	44.8
Powdered sugar	38	14.2	38	14.2	38	14.2	38	14.2
Extra fine iodised sea salt	0.1	0.04	0.1	0.04	0.1	0.04	0.1	0.04
Superior white wheat flour type 000	100	37.4	90	33.6	85	31.8	80	29.9
Sunflower liquid lecithin	1.7	0.6	1.7	0.6	1.7	0.6	1.7	0.6
Refined sunflower oil	7.8	2.9	7.8	2.9	7.8	2.9	7.8	2.9
Rosehip powder	-	-	10	3.7	15	5.6	20	7.5
Total	267.6	100	267.6	100	267.6	100	267.6	100
Substitution level		0		10		15		20

**Table 2:** Proximate composition, energy value, and pH of rosehip powder, batters, and waffle cones.

Parameter/Energy Value	Rp	WCc	WC3.7%rp	WC5.6%rp	WC7.5%rp
Moisture (%)	7.13 ± 0.114	1.94 ± 0.023 ^c^	2.06 ± 0.026 ^bc^	2.12 ± 0.064 ^b^	2.43 ± 0.007 ^a^
Protein (%)	6.98 ± 0.004	14.34 ± 0.499 ^a^	10.83 ± 0.509 ^b^	10.12 ± 0.003 ^b^	9.76 ± 0.0 ^b^
Fat (%)	3.31 ± 0.004	4.64 ± 0.005 ^b^	5.22 ± 0.164 ^ab^	5.23 ± 0.191 ^ab^	5.79 ± 0.249 ^a^
Ash (%)	3.31 ± 0.081	0.62 ± 0.039 ^c^	0.81 ± 0.017 ^b^	0.88 ± 0.018 ^ab^	0.97 ± 0.014 ^a^
Total carbohydrate (%)	79.27 ± 0.242	78.46 ± 0.510 ^b^	81.05 ± 0.228 ^a^	81.08 ± 0.717 ^a^	81.64 ± 0.143 ^a^
Crude fibre (%)	37.70 ± 0.042	1.58 ± 0.028 ^c^	3.41 ± 0.035 ^b^	4.65 ± 0.014 ^a^	4.83 ± 0.184 ^a^
Energy value (kcal/100 g)	375 ± 0.562	413 ± 0.089 ^a^	414 ± 1.141 ^a^	415 ± 0.644 ^a^	415 ± 1.333 ^a^
pH	3.73 ± 0.007	5.57 ± 0.0 ^a^	4.90 ± 0.021 ^b^	4.79 ± 0.0 ^c^	4.73 ± 0.0 ^d^
	Parameter	Bc	B3.7%rp	B5.6%rp	B7.5%rp
	pH	5.70 ± 0.007 ^a^	4.56 ± 0.0 ^b^	4.42 ± 0.021 ^c^	4.35 ± 0.021 ^d^

Data are presented as mean ± standard deviation of three measurements. Different lowercase letters within a row indicate significant differences between batters and waffle cone formulations (*p* < 0.05, Tukey’s test).

**Table 3:** Colour attributes of rosehip powder, batters, and waffle cones.

Parameter	Rp	Batter				Waffle Cone			
		BCc	BC3.7%rp	BC5.6%rp	BC7.5%rp	WCc	WC3.7%rp	WC5.6%rp	WC7.5%rp
	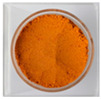	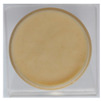	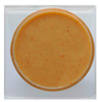	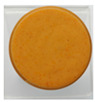	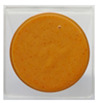	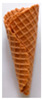	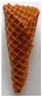	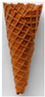	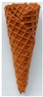
L*	62.02 ± 0.287	72.85 ± 0.678 ^a^	61.68 ± 0.915 ^b^	58.57 ± 0.883 ^c^	57.64 ± 0.618 ^d^	45.78 ± 2.406 ^a^	37.15 ± 1.800 ^b^	34.84 ± 1.861 ^c^	32.93 ± 1.446 ^c^
a*	17.75 ± 0.344	2.36 ± 0.145 ^d^	9.51 ± 0.275 ^c^	10.43 ± 0.367 ^b^	12.17 ± 0.281 ^a^	13.50 ± 0.875 ^a^	13.76 ± 0.889 ^a^	13.82 ± 0.419 ^a^	14.16 ± 0.778 ^a^
b*	31.06 ± 0.695	13.32 ± 0.276 ^d^	23.48 ± 0.536 ^c^	25.89 ± 0.522 ^b^	27.57 ± 0.366 ^a^	25.71 ± 0.995 ^a^	22.47 ± 1.232 ^b^	20.66 ± 1.288 ^c^	20.18 ± 1.358 ^c^
h*	1.05 ± 0.005	1.40 ± 0.009 ^a^	1.19 ± 0.006 ^b^	1.19 ± 0.007 ^b^	1.16 ± 0.005 ^c^	1.08 ± 0.012 ^a^	1.01 ± 0.016 ^b^	0.99 ± 0.016 ^c^	0.97 ± 0.012 ^d^
C*	35.77 ± 0.759	13.53 ± 0.286 ^d^	25.33 ± 0.585 ^c^	27.91 ± 0.606 ^b^	30.14 ± 0.433 ^a^	29.19 ± 1.020 ^a^	26.56 ± 1.397 ^b^	24.69 ± 1.508 ^c^	24.42 ± 1.597 ^c^
ΔE* vs. control		-	16.75	20.71	23.05	-	9.48	12.26	14.15

Data are presented as mean ± standard deviation of twelve measurements. Different lowercase letters within a row indicate significant differences between batters and waffle cone formulations (*p* < 0.05, Tukey’s test); ΔE* = 0.0–0.5, a colour difference at the trace level; ΔE* > 0.5 ≤ 1.5, the colour difference is slight; ΔE* > 1.5 ≤ 3.0, the colour difference is noticeable; ΔE* > 3.0 ≤ 6.0, the colour difference is appreciable; ΔE* > 6.0 ≤ 12.0, the colour difference is large; ΔE* > 12.0, the colour difference is obvious.

**Table 4:** Scores for waffle cone attributes following the Hedonic test and calculated acceptance rates.

Formulation	Sensory Attributes							Acceptance Rate (%)
	Appearance	Colour	Odour	Taste	Texture	Overall Acceptability	Overall Score	
WCc	8.2 ± 0.800 ^a^	8.1 ± 1.013 ^a^	7.8 ± 0.970 ^a^	8.1 ± 0.881 ^a^	8.4 ± 0.816 ^a^	8.1 ± 0.759 ^a^	8.1 ± 0.707 ^a^	90
WC3.7%rp	8.5 ± 0.823 ^a^	8.4 ± 0.707 ^a^	8.4 ± 0.638 ^a^	8.3 ± 0.792 ^a^	8.4 ± 0.651 ^a^	8.5 ± 0.714 ^a^	8.4 ± 0.603 ^a^	93
WC5.6%rp	8.5 ± 0.770 ^a^	8.2 ± 0.831 ^a^	8.3 ± 0.792 ^a^	8.3 ± 0.852 ^a^	8.4 ± 0.768 ^a^	8.4 ± 0.764 ^a^	8.4 ± 0.669 ^a^	93
WC7.5%	8.4 ± 0.860 ^a^	8.2 ± 0.746 ^a^	8.3 ± 0.891 ^a^	8.3 ± 0.936 ^a^	8.3 ± 0.852 ^a^	8.3 ± 0.852 ^a^	8.3 ± 0.651 ^a^	92

Data are expressed as mean ± standard deviation values of twenty-five responses. Different lowercase letters within a column indicate significant differences between waffle cone formulations (*p* < 0.05, Tukey’s test).

**Table 5:** Scores for waffle cone attributes following the descriptive evaluation test.

Formulation	Texture Attributes and Aroma			
	Fracturability	Fruity Aroma	Hardness	Crispness
WCc	4.0 ± 0.841 ^a^	1.6 ± 0.821 ^b^	2.8 ± 0.831 ^a^	3.9 ± 1.013 ^a^
WC3.7%rp	4.0 ± 0.978 ^a^	3.1 ± 1.115 ^a^	3.0 ± 0.866 ^a^	4.0 ± 0.841 ^a^
WC5.6%rp	3.8 ± 0.764 ^a^	3.1 ± 0.862 ^a^	3.2 ± 0.943 ^a^	4.0 ± 0.841 ^a^
WC7.5%	3.8 ± 1.179 ^a^	3.6 ± 0.870 ^a^	3.2 ± 0.866 ^a^	4.0 ± 0.889 ^a^

Data are expressed as mean ± standard deviation values of twenty-five responses. Different lowercase letters within a column indicate significant differences between waffle cone formulations (*p* < 0.05, Tukey’s test).

**Table 6:** Texture properties of waffle cones.

Formulation	Texture Attributes	
	Hardness (N)	Fracturability (Number of Fractures)
WCc	2.42 ± 0.306 ^a^	6 ± 1.155 ^a^
WC3.7%rp	2.31 ± 0.110 ^a^	7 ± 1.528 ^a^
WC5.6%rp	2.59 ± 0.095 ^a^	7 ± 1.000 ^a^
WC7.5%	2.83 ± 0.220 ^a^	8 ± 1.528 ^a^

Data are presented as mean ± standard deviation of three measurements. Different lowercase letters within a column indicate significant differences between batters and waffle cone formulations (*p* < 0.05, Tukey’s test).

**Table 7:** Techno-functional properties of rosehip powder and waffle cones.

Parameter	Rp	WCc	WC3.7%rp	WC5.6%rp	WC7.5%rp
WHC (g/g)	1.84 ± 0.056	3.11 ± 0.030 ^a^	3.08 ± 0.106 ^a^	3.02 ± 0.031 ^a^	2.64 ± 0.049 ^b^
SC (mL/g)	3.74 ± 0.347	4.98 ± 0.004 ^a^	4.48 ± 0.002 ^ab^	4.23 ± 0.357 ^ab^	4.23 ± 0.357 ^b^
OHC (g/g)	1.07 ± 0.079	0.93 ± 0.031 ^b^	0.96 ± 0.031 ^b^	1.05 ± 0.021 ^ab^	1.19 ± 0.062 ^a^
Solubility (%)	73.61 ± 0.700	75.33 ± 0.331 ^a^	74.15 ± 0.957 ^a^	73.93 ± 1.178 ^a^	72.65 ± 1.811 ^a^

Data are presented as mean ± standard deviation of three measurements. Different lowercase letters within a row indicate significant differences between batters and waffle cone formulations (*p* < 0.05, Tukey’s test).

**Table 8:** Content of polyphenolic compounds (µg/g) in rosehip powder and waffle cones.

Crt. No.	Compound	Class/ Subclass	Rp	WCc	WC3.7%rp	WC5.6%rp	WC7.5%rp
1	2-Hydroxybenzoic acid	PAs/HBAs	1245.87 ± 48.925	75.46 ± 4.426 ^d^	273.92 ± 5.857 ^c^	339.45 ± 11.857 ^b^	377.74 ± 10.191 ^a^
2	Procyanidin dimer B1	FVs/FVals	548.05 ± 16.493	n.d.	210.43 ± 9.358 ^c^	379.17 ± 1.711 ^b^	488.79 ± 10.657 ^a^
3	Protocatechuic acid	PAs/HBAs	581.09 ± 11.380	237.19 ± 4.481 ^d^	270.63 ± 5.165 ^c^	320.18 ± 2.402 ^b^	379.67 ± 7.089 ^a^
4	Procyanidin dimer B3	FVs/FVals	925.70 ± 29.031	n.d.	n.d.	n.d.	n.d.
5	Cyanidin 3-*O*-glucoside	FVs/ACNs	32.10 ± 0.517	n.d.	n.d.	n.d.	n.d.
6	Procyanidin dimer B2	FVs/FVals	518.62 ± 9.163	n.d.	n.d.	n.d.	n.d.
7	Chlorogenic acid	PAs/HCAs	n.d.	88.04 ± 1.098 ^a^	77.07 ± 0.026 ^b^	70.87 ± 0.351 ^c^	65.57 ± 0.597 ^d^
8	Procyanidin trimer C2	FVs/FVals	745.02 ± 7.167	n.d.	246.45 ± 1.022 ^c^	320.18 ± 2.402 ^b^	409.99 ± 5.146 ^a^
9	Catechin	FVs/FVals	1031.03 ± 43.230	n.d.	349.02 ± 0.813 ^b^	464.73 ± 40.088 ^a^	501.33 ± 9.640 ^a^
10	Procyanidin trimer C1	FVs/FVals	459.83 ± 2.570	n.d.	177.30 ± 5.086 ^c^	231.39 ± 1.440 ^b^	275.84 ± 6.913 ^a^
11	Ellagic acid glucoside	PAs/HBAs	131.97 ± 7.368	n.d.	14.40 ± 0.589 ^c^	16.91 ± 0.395 ^b^	21.61 ± 0.554 ^a^
12	Vanillin	PAs/HBAs	308.77 ± 13.740	n.d.	10.75 ± 0.036 ^b^	20.12 ± 2.286 ^a^	24.25 ± 0.507 ^a^
13	Quercetin 3-*O*-glucoside	FVs/Fvols	243.76 ± 3.138	n.d.	23.70 ± 0.580 ^c^	32.44 ± 0.268 ^b^	39.02 ± 1.852 ^a^
14	Ellagic acid	PAs/HBAs	251.09 ± 6.326	n.d.	n.d.	n.d.	n.d.
15	Quercetin 3-*O*-glucuronide	FVs/Fvols	73.12 ± 2.090	n.d.	n.d.	14.60 ± 0.014 ^a^	15.27 ± 0.502 ^a^
16	Kaempferol 3-*O*-glucoside	FVs/Fvols	117.11 ± 1.203	n.d.	14.93 ± 0.454 ^c^	17.10 ± 0.253 ^b^	19.38 ± 0.126 ^a^
17	5-Sinapoylquinic acid	PAs/HCAs	83.92 ± 2.036	n.d.	16.79 ± 0.718 ^b^	18.43 ± 0.254 ^ab^	20.71 ± 0.831 ^a^
18	Syringic acid	PAs/HBAs	62.68 ± 26.083	n.d.	25.71 ± 0.821 ^c^	32.44 ± 0.739 ^b^	37.69 ± 0.896 ^a^
19	Tiliroside	FVs/Fvols	225.35 ± 32.122	n.d.	21.17 ± 0.781 ^c^	28.27 ± 0.500 ^b^	38.85 ± 1.149 ^a^
	Total content		7585.05	400.70	1732.26	2306.30	2715.69

Data are presented as mean ± standard deviation of three measurements. Different lowercase letters within a row indicate significant differences between waffle cone formulations (*p* < 0.05, Tukey’s test).

**Table 9:** Content of carotenoid compounds (µg/g) in rosehip powder and waffle cones.

Crt. No.	Compound	Rp	WCc	WC3.7%rp	WC5.6%rp	WC7.5%rp
1	Lutein	1.50 ± 0.048	n.d.	n.d.	0.03 ± 0.007 ^b^	0.14 ± 0.014 ^a^
2	Lycopene	31.95 ± 0.164	n.d.	4.17 ± 0.083 ^c^	5.06 ± 0.004 ^b^	7.61 ± 0.039 ^a^
3	*β*-Carotene	75.59 ± 0.191	n.d.	2.69 ± 0.074 ^c^	4.34 ± 0.222 ^b^	6.53 ± 0.326 ^a^
	Total content	109.04	n.d.	6.86	9.43	14.28

Data are presented as mean ± standard deviation of three measurements. Different lowercase letters within a row indicate significant differences between waffle cone formulations (*p* < 0.05, Tukey’s test).

**Table 10:** Raw material costs of waffle cones.

Crt. No.	Ingredient	Inputs Used per Production							
		WCc		WC3.7%rp		WC5.6%rp		WC7.5%rp	
		Quantity (kg)	Cost (EUR) *	Quantity (kg)	Cost (EUR) *	Quantity (kg)	Cost (EUR) *	Quantity (kg)	Cost (EUR) *
1	Water	120	0.28	120	0.28	120	0.28	120	0.28
2	Powdered sugar	38	190.89	38	190.89	38	190.89	38	190.89
3	Extra fine iodised sea salt	0.1	0.08	0.1	0.08	0.1	0.08	0.1	0.08
4	Superior white wheat flour type 000	100	46.22	90	41.59	85	39.28	80	36.97
5	Sunflower liquid lecithin	1.7	70.03	1.7	70.03	1.7	70.03	1.7	70.03
6	Refined sunflower oil	7.8	11.52	7.8	11.52	7.8	11.52	7.8	11.52
7	Rosehip powder	-	-	10	30.12	15	45.18	20	60.24
Total (kg)/Raw material cost per batch (EUR)		268	319.01	268	344.51	268	357.26	268	370.01
Raw material cost per waffle cone (EUR)		-	0.053	-	0.057	-	0.060	-	0.062

* Based on costs available during the summer of 2024.

## Data Availability

The original contributions presented in this study are included in the article/supplementary material. Further inquiries can be directed to the corresponding author.

## Supplementary Materials

The following supporting information can be downloaded at https://www.mdpi.com/article/10.3390/foods14010090/s1. Table S1: Operational costs for producing 1 kg of rosehip waste powder.

## Abbreviations

WCc	control waffle cone
WC3.7%rp	waffle cones formulated with 3.7% rosehip powder
WC5.6%rp	waffle cones formulated with 5.6% rosehip powder
WC7.5%rp	waffle cones formulated with 7.5% rosehip powder
Bc	batter for control waffle cone
B3.7%rp	batter for waffle cones formulated with 3.7% rosehip powder
B5.6%rp	batter for waffle cones formulated with 5.6% rosehip powder
B7.5%rp	batter for waffle cones formulated with 7.5% rosehip powder
Rp	rosehip powder
*L**	lightness
*a**	redness
*b**	yellowness
*h**	hue angle
*C**	chroma
Δ*E**	total colour difference
WCH	water-holding capacity
SC	swelling capacity
OHC	oil-holding capacity
PAs	phenolic acids
FVs	flavonoids
HBAs	hydroxybenzoic acids
HCAs	hydroxycinnamic acids
FVals	flavanols
Fvols	flavonols
ACNs	anthocyanins
n.d.	not detected
