# Supplementary material for: Utilisation of Rosehip Waste Powder as a Functional Ingredient to Enrich Waffle Cones with Fibres, Polyphenols, and Carotenoids
Source: Foods. 2025 Jan 1;14(1):90. doi: 10.3390/foods14010090 (PMC11719886; doi:10.3390/foods14010090)
Supplement: Supplementary file 1 [file foods-14-00090-s001.zip › foods-3387285-supplementary.pdf]

foods

MDPI

Article

Utilisation of Rosehip Waste Powder as a Functional Ingredient to Enrich Waffle Cones with Fibres, Polyphenols, and Carotenoids

Alexandra Raluca Borşa (Bogdan) [1], Adriana Păucean [1], Melinda Fogarasi [1], Floricuța Ranga [2], Andrei Borșa [1], Anda Elena Tanislav [1], Vlad Mureșan [1] and Cristina Anamaria Semeniuc [1,*]

[1] Department of Food Engineering, University of Agricultural Sciences and Veterinary Medicine of Cluj-Napoca, 3-5 Calea Mănăştur, 400372 Cluj-Napoca, Romania; raluca.borsa@usamvcluj.ro (A.R.B.B.); adriana.paucean@usamvcluj.ro (A.P.); melinda.fogarasi@usamvcluj.ro (M.F.); andrei.borsa@usamvcluj.ro (A.B.); anda.tanislav@usamvcluj.ro (A.E.T.); vlad.muresan@usamvcluj.ro (V.M.)
[2] Department of Food Science, University of Agricultural Sciences and Veterinary Medicine of Cluj-Napoca, 3-5 Calea Mănăştur, 400372 Cluj-Napoca, Romania; floricutza_ro@yahoo.com (F.R.)
* Correspondence: cristina.semeniuc@usamvcluj.ro; Tel.: +40-264-596-384 (C.A.S.)

Table S1. Operational costs for producing 1 kg of rosehip waste powder.

Cost category	Justification	Cost of final product per kilogram (€/kg)
Acquisition of raw material	1.72 kg rosehip waste × 0.2 €/kg (incl. shipping)	0.344
Equipment cost	Small-capacity dehydrator (2,000 €) lifespan 5 years, processing 10 kg/day, 250 days/year<br>Daily equipment cost = 1.6 €; Yield = 58.1%; Equipment cost/kg = 1.6 €/5.81 kg	0.275
Labor	1000 € gross salary~ at 6 € per hour<br>1.5 h labour time × 6 €/h/5.81 kg final product	1.549
Energy	Power consumption × usage time/day × electricity cost ÷ final product quantity<br>1 kWh × 10 h × 0.2 €/kWh ÷ 5.81 kg	0.344
Packaging and maintenance	Estimated cost for packaging materials and equipment maintenance	0.500
**Total operational cost**	**Sum of all cost categories**	**3.012**
